# Supplementary material for: A multi-omics framework for survival mediation analysis of high-dimensional proteogenomic data
Source: PLoS Comput Biol. 2026 Apr 27;22(4):e1014217. doi: 10.1371/journal.pcbi.1014217 (PMC13138757; doi:10.1371/journal.pcbi.1014217)
Supplement: S1 Appendix — Detailed derivation of the penalized AFT model and MCP-penalized mediation model used for screening in Step 1. (PDF) [file pcbi.1014217.s001.pdf]

S1 Appendix

1 Penalized Outcome Model in Step 1

Suppose the outcome model penalization is applied separately for $\boldsymbol{X}$ or $\boldsymbol{M}$ when $\boldsymbol{\Phi}_i^\top = \boldsymbol{X}$ and $\boldsymbol{\theta} = \boldsymbol{\beta}_{\boldsymbol{X}}$, and when $\boldsymbol{\Phi}_i^\top = \boldsymbol{M}$ and $\boldsymbol{\theta} = \boldsymbol{\beta}_{\boldsymbol{M}}$, respectively.

A penalized Gehan-type estimator of $\boldsymbol{\theta}$ is constructed after some algebra,

$$\arg\min_{\boldsymbol{\theta}} \left\{ \frac{1}{n^2} \sum_{i=1}^{n} \sum_{j=1}^{n} \frac{\delta_i}{b} \Big[\log t_i - \log t_j - (\boldsymbol{\Phi}_i - \boldsymbol{\Phi}_j)^\top \boldsymbol{\theta} \Big]^- + \lambda g(\boldsymbol{\theta}) \right\},$$

where we denote $\{f\}^- = \max(-f, 0)$, $\lambda > 0$ as a user-specified tuning parameter, and $g(\boldsymbol{\theta})$ as a convex penalty function for all regression parameters of interest (*i.e.* proteomes and genes) in the outcome model. Currently, three penalty functions are implemented in penAFT R package: weighted sparse-group lasso [1], weighted elastic-net [2], and ridge [3]. The weighted sparse-group lasso penalty is used when function g is defined as

$$\gamma \left\| \boldsymbol{w} \circ \boldsymbol{\theta} \right\|_1 + (1-\gamma) \sum_{l=1}^{G} v_l \left\| \boldsymbol{\theta}_{\mathcal{G}_l} \right\|_2 ,$$

where $\gamma \in [0,1]$ is a tuning parameter, $\boldsymbol{w}$ and v_l non-negative weights for $\boldsymbol{\theta}_{\mathcal{G}_l}$, $l = 1, \ldots, G$ partitions of all regression parameters.

The weighted elastic-net is similarly expressed with L_1 and L_2 regularizations but without v_l-contributed weights as

$$\gamma \left\| \boldsymbol{w} \circ \boldsymbol{\theta} \right\|_1 + \frac{1-\gamma}{2} \left\| \boldsymbol{\theta} \right\|_2^2 . \tag{1}$$

The g penalty function reduces to the L_1 term when $\gamma = 0$ in Eq 1, which is known as the ridge penalization.

2 Penalized Mediation Model in Step 1

The MCP estimates are obtained by

$$\arg\min_{\boldsymbol{\alpha}_{\boldsymbol{X}}} \left\{ \frac{1}{2n} \sum_{i=1}^{n} \Big[M_{ki} - \boldsymbol{X}_i^\top \boldsymbol{\alpha}_{\boldsymbol{X}}^k \Big]^2 + \sum_{k=1}^{K} h_{\lambda,\tau}(\boldsymbol{\alpha}_{\boldsymbol{X}}^k) \right\},$$

where $h_{c,\tau}(\boldsymbol{\alpha}_{\boldsymbol{X}}^k)$ is MCP function, defined as

$$h_{\lambda,\tau}(\boldsymbol{\alpha}_{\boldsymbol{X}}^k) = \begin{cases} \lambda |\boldsymbol{\alpha}_{\boldsymbol{X}}^k| - \frac{(|\boldsymbol{\alpha}_{\boldsymbol{X}}^k|)^2}{2\tau}, & |\boldsymbol{\alpha}_{\boldsymbol{X}}^k| \leq \tau\lambda \\ \frac{1}{2}\tau\lambda^2 & |\boldsymbol{\alpha}_{\boldsymbol{X}}^k| > \tau\lambda \end{cases},$$

where $\lambda \geq 0$ is the regularization parameter and $\tau > 1$ is the tuning parameter. The ncvreg R package [4] was used fit the model using the MCP penalty.

References

[1] Simon N, Friedman J, Hastie T, Tibshirani R. A sparse-group lasso. J Comput Graph Stat. 2013;22:231–245.

[2] Zou H, Zhang HH. On the adaptive elastic-net with a diverging number of parameters. Ann Stat. 2009;37:1733–1751.

[3] Hoerl AE, Kennard RW. Ridge regression: biased estimation for nonorthogonal problems. Technometrics. 2000;42:80–86.

[4] Breheny P, Huang J. Coordinate descent algorithms for nonconvex penalized regression, with applications to biological feature selection. Ann Appl Stat. 2011;5:232–253.
