# Supplementary material for: A multi-omics framework for survival mediation analysis of high-dimensional proteogenomic data
Source: PLoS Comput Biol. 2026 Apr 27;22(4):e1014217. doi: 10.1371/journal.pcbi.1014217 (PMC13138757; doi:10.1371/journal.pcbi.1014217)
Supplement: S1 Table — The default MCP penalized mediation model is compared with mediation models using elastic-net and Lasso penalties. (PDF) [file pcbi.1014217.s003.pdf]

S1 Table

S1 Table. Simulation results of the SMAHP with varying penalties in the mediation model. The default MCP penalized mediation model is compared with mediation models using elastic-net and Lasso penalties. Notably, the penalization for the AFT outcome model in Step 1 and the sure independence screening in Step 2 remain consistent across all scenarios. The data were simulated with a censoring rate of 25%.

Scenario	p	k	n	Method	Power	FDR	Average Computational Time (in minutes)
I	50	100	200	Pen^1 + SIS	0.9853	0.0313	1.72
				Pen^2 + SIS	0.9855	0.0292	1.03
				Pen^3 + SIS	0.9855	0.0289	1.60
			400	Pen^1 + SIS	1.0000	0.0337	4.51
				Pen^2 + SIS	1.0000	0.0333	6.92
				Pen^3 + SIS	1.0000	0.0323	6.32
II	50	200	200	Pen^1 + SIS	0.9780	0.0362	1.32
				Pen^2 + SIS	0.9780	0.0365	1.74
				Pen^3 + SIS	0.9778	0.0357	1.74
			400	Pen^1 + SIS	0.9995	0.0290	4.89
				Pen^2 + SIS	0.9998	0.0296	4.51
				Pen^3 + SIS	0.9998	0.0306	6.49
III	100	100	200	Pen^1 + SIS	0.8296	0.0114	1.63
				Pen^2 + SIS	0.8274	0.0126	1.11
				Pen^3 + SIS	0.8273	0.0126	1.10
			400	Pen^1 + SIS	0.9899	0.0244	5.05
				Pen^2 + SIS	0.9898	0.0224	4.51
				Pen^3 + SIS	0.9899	0.0227	4.62
IV	100	200	200	Pen^1 + SIS	0.8380	0.0192	2.89
				Pen^2 + SIS	0.8356	0.0182	1.89
				Pen^3 + SIS	0.8369	0.0190	1.29
			400	Pen^1 + SIS	0.9960	0.0205	5.33
				Pen^2 + SIS	0.9961	0.0202	6.43
				Pen^3 + SIS	0.9964	0.0202	4.71

Abbreviations: FDR, false discovery rate.
Pen^1 = MCP penalized mediation model and penalized AFT outcome model in Step 1; Pen^2 = elastic-net penalized mediation model and penalized AFT outcome model in Step 1; Pen^3 = Lasso penalized mediation model and penalized AFT outcome model in Step 1; SIS = sure independence screening in Step 2
n = sample size; p = number of genes (exposures); k = number of proteins (mediators)
