# Supplementary material for: A multi-omics framework for survival mediation analysis of high-dimensional proteogenomic data
Source: PLoS Comput Biol. 2026 Apr 27;22(4):e1014217. doi: 10.1371/journal.pcbi.1014217 (PMC13138757; doi:10.1371/journal.pcbi.1014217)
Supplement: S2 Table — SMAHP was assessed with correlated gene and protein structures. (PDF) [file pcbi.1014217.s004.pdf]

S2 Table

S2 Table. Simulation results for the SMAHP model (penalization + SIS) with correlated gene and protein structures. Data were simulated under censoring rates of 25%.

p	k	n	ρ_X	ρ_M	Power	FDR
50	100	200	0.4	0.4	0.8175	0.0202
		400	0.4	0.4	0.9605	0.0122
50	200	200	0.4	0.4	0.8065	0.0225
		400	0.4	0.4	0.9320	0.0065
100	100	200	0.4	0.4	0.6821	0.0272
		400	0.4	0.4	0.8741	0.0035
100	200	200	0.4	0.4	0.6191	0.0307
		400	0.4	0.4	0.8201	0.0038

Abbreviations: FDR, false discovery rate.
n = sample size; p = number of genes (exposures); k = number of proteins (mediators); ρ_X = correlation among genes; ρ_M = correlation among proteins.
