# Supplementary material for: A multi-omics framework for survival mediation analysis of high-dimensional proteogenomic data
Source: PLoS Comput Biol. 2026 Apr 27;22(4):e1014217. doi: 10.1371/journal.pcbi.1014217 (PMC13138757; doi:10.1371/journal.pcbi.1014217)
Supplement: S3 Table — SMAHP was further evaluated under this alternative exposure distribution. (PDF) [file pcbi.1014217.s005.pdf]

## S3 Table

S3 Table. Simulation results of the SMAHP where exposures were generated from a negative binomial distribution, with a censoring rate of 25%.

| Scenario | $p$ | $k$ | $n$ | Power | FDR |
|---|---|---|---|---|---|
| I | 50 | 100 | 200 | 0.9350 | 0.0190 |
| | | | 400 | 0.9938 | 0.0178 |
| II | 50 | 200 | 200 | 0.9180 | 0.0194 |
| | | | 400 | 0.9860 | 0.0172 |
| III | 100 | 100 | 200 | 0.8220 | 0.0147 |
| | | | 400 | 0.9900 | 0.0156 |
| IV | 100 | 200 | 200 | 0.7651 | 0.0346 |
| | | | 400 | 0.9741 | 0.0149 |

Abbreviations: FDR, false discovery rate.
$n$ = sample size; $p$ = number of genes (exposures); $k$ = number of proteins (mediators)
