# Supplementary material for: A multi-omics framework for survival mediation analysis of high-dimensional proteogenomic data
Source: PLoS Comput Biol. 2026 Apr 27;22(4):e1014217. doi: 10.1371/journal.pcbi.1014217 (PMC13138757; doi:10.1371/journal.pcbi.1014217)
Supplement: S4 Table — We further evaluated the performance of SMAHP under this setting with outliers. (PDF) [file pcbi.1014217.s006.pdf]

S4 Table

S4 Table. Simulation results of the SMAHP in the presence of outliers, with censoring rates of 25%.

Scenario	p	k	n	Power	FDR
I	50	100	200	0.9573	0.0160
			400	0.9963	0.0111
II	50	200	200	0.9280	0.0171
			400	0.9818	0.0125
III	100	100	200	0.7896	0.0241
			400	0.9736	0.0064
IV	100	200	200	0.7596	0.0285
			400	0.9490	0.0051

Abbreviations: FDR, false discovery rate.
n = sample size; p = number of genes (exposures); k = number of proteins (mediators)
