# Supplementary material for: A multi-omics framework for survival mediation analysis of high-dimensional proteogenomic data
Source: PLoS Comput Biol. 2026 Apr 27;22(4):e1014217. doi: 10.1371/journal.pcbi.1014217 (PMC13138757; doi:10.1371/journal.pcbi.1014217)
Supplement: S5 Table — SMAHP was evaluated under Gamma residual distributions. (PDF) [file pcbi.1014217.s007.pdf]

S5 Table

S5 Table. Simulation results of SMAHP under a Gamma error distribution with censoring rates of 25%.

Scenario	p	k	n	Power	FDR
I	50	100	200	0.9758	0.0246
			400	0.9950	0.0282
II	50	200	200	0.9825	0.0254
			400	0.9988	0.0127
III	100	100	200	0.8364	0.0163
			400	0.9964	0.0152
IV	100	200	200	0.8211	0.0210
			400	0.9958	0.0134

Abbreviations: FDR, false discovery rate.
n = sample size; p = number of genes (exposures); k = number of proteins (mediators)
