# Supplementary material for: A multi-omics framework for survival mediation analysis of high-dimensional proteogenomic data
Source: PLoS Comput Biol. 2026 Apr 27;22(4):e1014217. doi: 10.1371/journal.pcbi.1014217 (PMC13138757; doi:10.1371/journal.pcbi.1014217)
Supplement: S6 Table — SMAHP was evaluated under logistic residual distributions. (PDF) [file pcbi.1014217.s008.pdf]

## S6 Table

S6 Table. Simulation results of SMAHP under a logistic error distribution with censoring rates of 25%.

| Scenario | $p$ | $k$ | $n$ | Power | FDR |
|---|---|---|---|---|---|
| I | 50 | 100 | 200 | 0.9770 | 0.0352 |
| | | | 400 | 1.0000 | 0.0240 |
| II | 50 | 200 | 200 | 0.9625 | 0.0388 |
| | | | 400 | 1.0000 | 0.0297 |
| III | 100 | 100 | 200 | 0.8304 | 0.0290 |
| | | | 400 | 0.9943 | 0.0199 |
| IV | 100 | 200 | 200 | 0.8194 | 0.0279 |
| | | | 400 | 0.9876 | 0.0279 |

Abbreviations: FDR, false discovery rate.
$n$ = sample size; $p$ = number of genes (exposures); $k$ = number of proteins (mediators)
