# Supplementary material for: A multi-omics framework for survival mediation analysis of high-dimensional proteogenomic data
Source: PLoS Comput Biol. 2026 Apr 27;22(4):e1014217. doi: 10.1371/journal.pcbi.1014217 (PMC13138757; doi:10.1371/journal.pcbi.1014217)
Supplement: Attachment — Submitted filename: plos_reviewer_commetns.docx [file pcbi.1014217.s009.docx]

Ahn et al. perform an ambitious computational study of importance, particularly as multi-omic and high-dimensional biological data become increasingly available. The computational problem that the paper is trying to solve is clearly laid out, and the solution rationale is clear and logical. Overall, it is a strong study that has the potential to make an advancement in the field of causal mediation analysis. Prior to publication, however, there are some important gaps that should be addressed.

Major comments

1. Line 95-96: This assumption is likely biologically violated as many RNA transcripts will be correlated between one another. Prior to the use of SMAHP, it appears that a univariate AFT model is fit to each gene and protein for pre-screening. Does this univariate AFT account for or handle the underlying correlation structure? If correlated markers are chosen, does this impact any downstream interpretation of the results?

2. Lines 167-169: How was the underlying correlation structure assessed for the selected exposure genes and mediator proteins, such as correlation among selected exposure genes, among selected mediator proteins, or between selected exposure/mediator? If this does not need to be addressed, why?

3. Line 175: Was this model run with any additional covariates, if the data was available? For example, sex is another important covariate that should not inflate model complexity beyond what the data can support.

4. The use of different censoring rates is a key problem that the authors mentioned they were solving, in addition to being presented in Tables 1 – 3. There is little discussion, however, of how censoring rates might impact study design. This could be expanded further in the discussion. How does an understanding of censoring rate for a given biological dataset help researchers who are interested in using this tool to design their study? What modifications do they need to make in the case of a high or low censoring rate?

5. RNA-seq data (and omics data in general) may be more labile than the distribution used for the exposure variables in the simulation study. Are the results consistent if the exposure is sampled from a different distribution, such as a negative binomial distribution? If challenging to test this, it may be warranted to expand the limitations section of the Discussion beyond a nonparametric AFT model but also to alternative exposure and mediator distributions.

6. The authors identify a positive direct effect and negative indirect effect through the mediator, but it is not expanded further. What could this mean biologically?

7. What was the censoring rate in the CPTAC HNSCC data, if available (or if I missed it)? This is important to discuss because the simulation studies showed us that SMAHP may require higher sample sizes at higher censoring rates.

8. The limitations section of the Discussion could be expanded as mentioned above. Additionally, other limitations to consider discussing are a lack of validation dataset (difficult to obtain likely for this cancer type, but still important) and lack of application of the method to another multi-omics, high-dimensional dataset.

Minor comments

1. Lines 65-69: These sentences could benefit from more precision. I believe the point is that proteogenomic studies are clinically relevant (evidenced by references 15-21) but are understudied in high-dimensional causal mediation analysis. If that understanding is correct, please rephrase these sentences to make that point more clear.

2. Line 79: There is a typo in this sentence as “accelerated time failure” should instead be written as “accelerated failure time”.

3. The naïve model presented in Tables 1 – 3 seems like a negative control. You could consider listing it first in the tables, followed by the benchmarking method (SIS+SIS?), and then the new method. The superscript in “Pen” in the table is also unclear. I believe it stands for “Penalizations”, but making that connection more clear would be helpful. Lastly, making the connection that SMAHP is the Pen + SIS model more clear would be helpful.

4. Line 152: While RNA expression is correlated with germline/somatic mutations, I would be cautious calling the RNA-seq data “genetic alterations”.

5. Line 183-185: I think this sentence should be re-visited as this statement is quite strong. An alternative explanation is that these individuals could be HPV-negative because they are not infected. As written, the conclusion appears too strong without additional evidence.

6. Lines 178-187 seem like they belong in the Discussion.

7. Line 211: Consider rephrasing to “Our simulation study” for clarity.

8. Lines 239 through 244 seem out of place and should be placed elsewhere in the Discussion.
