# Supplementary material for: A multi-omics framework for survival mediation analysis of high-dimensional proteogenomic data
Source: PLoS Comput Biol. 2026 Apr 27;22(4):e1014217. doi: 10.1371/journal.pcbi.1014217 (PMC13138757; doi:10.1371/journal.pcbi.1014217)
Supplement: Attachment — Submitted filename: Response_to_Reviewers.docx [file pcbi.1014217.s011.docx]

**Dear Editor:**

We thank the reviewers for their valuable comments/feedback, and we have carefully revised our manuscript accordingly. We believe that our manuscript has been substantially improved by responding to their comments. Please see our point-by-point responses below.

**Reviewer #1**

Ahn et al. perform an ambitious computational study of importance, particularly as multi-omic and high-dimensional biological data become increasingly available. The computational problem that the paper is trying to solve is clearly laid out, and the solution rationale is clear and logical. Overall, it is a strong study that has the potential to make an advancement in the field of causal mediation analysis. Prior to publication, however, there are some important gaps that should be addressed.

**Authors’ response**: Thank you for the appraisal.

Major comments

1. Line 95-96: This assumption is likely biologically violated as many RNA transcripts will be correlated between one another. Prior to the use of SMAHP, it appears that a univariate AFT model is fit to each gene and protein for pre-screening. Does this univariate AFT account for or handle the underlying correlation structure? If correlated markers are chosen, does this impact any downstream interpretation of the results?

**Authors’ response**: Thank you for this thoughtful comment. Lines 95-96 pertain specifically to the data generation mechanism used in our simulations. Regarding univariate marginal screening, it has become one of the common practices^1, 2, 3^ to use univariate screening (e.g., the sure independence screening (SIS))^4^ to pre-screen mediators to reduce dimensionality in high dimensional mediation analyses. The univariate AFT models do not explicitly account for the correlation structure among genes or proteins, and correlated markers may therefore be retained after the screening step. To assess the impact of such correlation, we conducted additional simulation studies with correlated gene and protein structures where the correlation of the proteins are set to be 0.4 which is around the third quartile of magnitudes of observed correlations among the top 200 selected proteins and the correlation of the genes is also set to be 0.4 which is much more than third quartile of magnitudes of observed correlations among the top 100 selected genes. So the selected correlations are at the higher side according to the observed data. The results indicate that the performance of the proposed method remains consistent under these correlated settings. These additional simulations are now included as part of the sensitivity analyses (S2 Table). The results demonstrate that the proposed method remains stable and yields consistent performance even when correlation is present among predictors.

We have added “Prior to the application of the SMAHP, a univariable AFT model was fitted to each gene and protein separately for pre-screening purposes **in this ultra high-dimensional data**”

References:

[1] Liu L, Zhang H, Zheng Y, Gao T, Zheng C, Zhang K, Hou L, Liu L. High-dimensional mediation analysis for longitudinal mediators and survival outcomes. Brief Bioinform. 2025 May 1;26(3):bbaf206

[2] Zhang H, Hou L, Liu L. A Review of High-Dimensional Mediation Analyses in DNA Methylation Studies. Methods Mol Biol. 2022;2432:123-135

[3] Perera C, Zhang H, Zheng Y, Hou L, Qu A, Zheng C, Xie K, Liu L. HIMA2: high-dimensional mediation analysis and its application in epigenome-wide DNA methylation data. BMC Bioinformatics. 2022 Jul 25;23(1):296.

[4] Fan J and Lv J (2008), “Sure independence screening for ultrahigh dimensional feature space,” Journal of the Royal Statistical Society: Series B (Statistical Methodology), 70, 849–911.

2. Lines 167-169: How was the underlying correlation structure assessed for the selected exposure genes and mediator proteins, such as correlation among selected exposure genes, among selected mediator proteins, or between selected exposure/mediator? If this does not need to be addressed, why?

**Authors’ response**: We appreciate this comment. In the revised manuscript, we have extended the simulation studies to explicitly incorporate correlated gene and protein structures, which are included as part of the sensitivity analysis (S2 Table). The results indicate that the method maintains consistent performance. Please also see the response to the above comment 1 regarding correlated genes and proteins.

3. Line 175: Was this model run with any additional covariates, if the data was available? For example, sex is another important covariate that should not inflate model complexity beyond what the data can support.

**Authors’ response**: Thank you for this question. Yes, the model can include additional covariates in principle, but we tried to be conservative in the real study since the sample size (102) is limited. With the current model, there are totally 9 parameters (5 coefficient parameters, 2 intercept parameters and 2 standard deviations of the random errors) which gives us only 11 subjects per parameter. Plus, age has the strongest association with the outcome survival (p = 0.000016) which is why we only included age in the real data analysis.

4. The use of different censoring rates is a key problem that the authors mentioned they were solving, in addition to being presented in Tables 1 – 3. There is little discussion, however, of how censoring rates might impact study design. This could be expanded further in the discussion. How does an understanding of censoring rate for a given biological dataset help researchers who are interested in using this tool to design their study? What modifications do they need to make in the case of a high or low censoring rate?

**Authors’ response**: We appreciate this comment. We have expanded the Discussion section to describe how different censoring rates may impact study design when applying the proposed method. Specifically, we discuss how anticipated censoring rates can inform sample size planning, and how larger sample sizes may help mitigate the loss of power under high censoring conditions.

“These findings highlight the importance of the censoring rate when applying the proposed method and motivate further discussion of its implications for study design. Censoring rate is an important consideration when applying the proposed method and has direct implications for study design. Higher censoring rates generally reduce the effective information available for inference, which can lead to decreased statistical power. Our simulation results demonstrate that under high censoring conditions, such as 75%, increasing the sample size can substantially improve power while maintaining appropriate control of the false discovery rate.

From a practical perspective, when researchers anticipate a high censoring rate in a biological dataset, larger sample sizes may be required to achieve adequate performance. In contrast, for studies with relatively low censoring rates, the method performs well even with more moderate sample sizes. Therefore, understanding the expected censoring mechanism and rate in a given study can inform decisions regarding sample size planning and the feasibility of applying the proposed framework.”

5. RNA-seq data (and omics data in general) may be more labile than the distribution used for the exposure variables in the simulation study. Are the results consistent if the exposure is sampled from a different distribution, such as a negative binomial distribution? If challenging to test this, it may be warranted to expand the limitations section of the Discussion beyond a nonparametric AFT model but also to alternative exposure and mediator distributions.

**Authors’ response**: We thank the reviewer for this helpful suggestion. We conducted a sensitivity analysis with exposures sampled from a negative binomial distribution. The results are summarized in S3 Table which shows a stable performance. We have also updated our results section.

6. The authors identify a positive direct effect and negative indirect effect through the mediator, but it is not expanded further. What could this mean biologically?

**Authors’ response**: Thank you for this comment. We have added a brief clarification (in Application Study section and see below for your convenience) to explain how a positive direct effect together with a negative indirect effect can arise conceptually, noting that such a pattern may reflect multiple underlying pathways with opposing effects. This addition is intended to aid interpretation without overextending biological conclusions.

“Such a pattern may arise when the exposure influences survival through multiple pathways, with the mediator capturing an effect in the opposite direction to other pathways such as the direct pathway. This observation highlights the complexity of the underlying biological processes and is provided to aid interpretation of the estimated effects.”

7. What was the censoring rate in the CPTAC HNSCC data, if available (or if I missed it)? This is important to discuss because the simulation studies showed us that SMAHP may require higher sample sizes at higher censoring rates.

**Authors’ response**: The censoring rate was 67.6% for 102 participants that were eligible for the analysis (33 events). Please see our response to the above comment 4 on the discussion of censoring rate. In our revised manuscript, we have added the following information:

“…102 patients**, with a censoring rate of 67.6%.**”

8. The limitations section of the Discussion could be expanded as mentioned above. Additionally, other limitations to consider discussing are a lack of validation dataset (difficult to obtain likely for this cancer type, but still important) and lack of application of the method to another multi-omics, high-dimensional dataset.

**Authors’ response**: Thank you for this thoughtful comment. We have expanded the Discussion to address the limitation that higher sample sizes may be required under high censoring rates, based on our simulation results. In addition, we note that the proposed method was demonstrated using a single multi-omics, high-dimensional dataset, and an independent dataset was not available to serve as a traditional validation set. As a result, the same causal pathway may or may not be observed across different datasets, and differences in findings could reflect variations in cohort characteristics, such as age or ethnicity, rather than limitations of the proposed methodology.

We have added the following in Discussion section: “At present, the proposed method has been demonstrated using a single multi-omics, high-dimensional dataset, and an independent dataset was not available to serve as a traditional validation set. As a result, the same causal pathway may or may not be observed across different datasets, and differences in findings could reflect variations in cohort characteristics, such as age or ethnicity, rather than limitations of the proposed methodology. In future studies, we will try to validate the proposed approach using independent multi-omics datasets with larger sample sizes (if available).”

Minor comments

1. Lines 65-69: These sentences could benefit from more precision. I believe the point is that proteogenomic studies are clinically relevant (evidenced by references 15-21) but are understudied in high-dimensional causal mediation analysis. If that understanding is correct, please rephrase these sentences to make that point more clear.

**Authors’ response**: Thank you. We have updated the paragraph as the following:

“In addition, from a clinical perspective, most existing mediation methods have primarily been developed for analyses within a single omics layer. In contrast, a growing body of clinical proteogenomic studies [15-21] has demonstrated the clinical relevance of jointly analyzing genomic and proteomic data, highlighting their complementary roles and associations with disease phenotypes across diverse disease types. Despite this clinical importance, high-dimensional causal mediation methods that explicitly integrate proteomic and transcriptomic data remain relatively underdeveloped.

2. Line 79: There is a typo in this sentence as “accelerated time failure” should instead be written as “accelerated failure time”.

**Authors’ response**: Thank you for catching this typo. We have corrected it to “accelerated failure time.”

3. The naïve model presented in Tables 1 – 3 seems like a negative control. You could consider listing it first in the tables, followed by the benchmarking method (SIS+SIS?), and then the new method. The superscript in “Pen” in the table is also unclear. I believe it stands for “Penalizations”, but making that connection more clear would be helpful. Lastly, making the connection that SMAHP is the Pen + SIS model more clear would be helpful.

**Authors’ response**: We appreciate the reviewer’s suggestion. In the revised manuscript, we reordered Tables 1 to 3 to list the naïve model first, followed by the SIS-based model, and then the proposed SMAHP. We also clarified in the table footnotes that SMAHP refers to the penalized model with SIS, which is consistently indicated in the table titles as “SMAHP (penalization + SIS).”

4. Line 152: While RNA expression is correlated with germline/somatic mutations, I would be cautious calling the RNA-seq data “genetic alterations”.

**Authors’ response**: We have rephrased from “genetic alterations” to “transcriptomic and molecular changes” in our revised manuscript.

5. Line 183-185: I think this sentence should be re-visited as this statement is quite strong. An alternative explanation is that these individuals could be HPV-negative because they are not infected. As written, the conclusion appears too strong without additional evidence.

**Authors’ response**: We agree with your comment. We updated the sentence as the following to avoid overstatement:

“Interestingly, MalaCards indicates that LCE3E is associated with plantar warts, which are caused by HPV. **However, given that the study samples tested HPV-negative, this association should be interpreted with caution**.”

6. Lines 178-187 seem like they belong in the Discussion.

**Authors’ response**: Thank you for this comment. We acknowledge that Lines 178–187 include biological interpretation of the real-data findings. We intentionally retained this content in the current section, immediately following the presentation of the corresponding gene and protein results, to provide context at the point where the findings are introduced and to improve readability. The main Discussion section is reserved for broader interpretation and methodological considerations, and we therefore prefer to keep this content in its current location.

7. Line 211: Consider rephrasing to “Our simulation study” for clarity.

**Authors’ response**: Thank you. We have updated from “A simulation study” to “Our simulation study” in this resubmission.

8. Lines 239 through 244 seem out of place and should be placed elsewhere in the Discussion.

**Authors’ response**: Thank you for this comment. To improve clarity and readability, we have moved it up in Discussion section and also edited as follows:

“In particular, we explored alternative multiple hypothesis testing controls beyond the BH procedure, including the q-value method [51], Westfall-Young correction [60], and HDMT [59]. However, these approaches were not adopted, as they either resulted in worse performance or were incompatible with modeling high-dimensional exposures (the latter being the case for HDMT). Future methodological development that better accommodates high-dimensional exposure settings could make it possible to incorporate these alternative multiple testing approaches.”

**Reviewer #2**

Reviewer #2: This study presents a multi-omics survival mediation framework of significant methodological value, featuring a mathematically rigorous and innovative three-step workflow that effectively tackles high-dimensional data challenges and overcomes the limitations of traditional Cox models. This framework enables integrated genomic and proteomic analysis, addressing a critical gap in single-omics approaches and providing a generalizable paradigm that better reflects biological complexity. Furthermore, the model demonstrates high persuasiveness through comprehensive multi-scenario simulations, consistently showcasing superior statistical power and robust false discovery rate control compared to other methods.
However, the study has several critical issues that require further refinement.

**Authors’ response**: Thank you for the appraisal.

1. Validation of core model assumptions and clarification of applicability boundaries are insufficient: The outcome model of SMAHP is based on a parametric AFT model, assuming the error follows a normal distribution. However, the authors did not validate the validity of this distribution assumption in either simulated data or real-world data (CPTAC). If the actual data (e.g., survival time distribution) deviates from the normal assumption, it may lead to biased estimates of mediation effects. Additionally, the study did not investigate the applicability of the "proportional effect assumption" (linear associations between exposure and mediator, and between mediator and outcome). For example, it did not test for non-linear relationships between gene expression levels and protein abundances, nor did it explain the model’s robustness when interactions exist between variables. It is recommended to supplement residual distribution tests (e.g., Q-Q plots), non-linear relationship tests (e.g., adding quadratic terms), and clarify the limitations of the model when assumptions are violated.

**Authors’ response**: We thank the reviewer for this insightful comment. The outcome model in SMAHP is formulated within a parametric AFT framework, where the normality assumption pertains to the error term on the log-time scale. In practice, residual-based diagnostics such as Q-Q plots are difficult to interpret in the presence of censoring and do not provide clear or reliable guidance comparable to those in standard linear regression. Instead, we assessed the behavior of the proposed method primarily through additional simulation studies under different data-generating mechanisms in our main manuscript and supplementary tables. The additional simulation analyses using AFT models with two alternative residual distributions (Gamma and logistic error) are presented in S5-S6 Tables in Supplementary Materials document. The results showed consistent performance, with the method maintaining high power and controlling the FDR under low to moderate censoring rates. We have also added interpretation in Results section as well.

Regarding potential non-linear and interaction effects, while incorporating quadratic or interaction terms is possible in principle, doing so in a high-dimensional mediation setting with many candidate genes and proteins would substantially increase the number of terms to be evaluated and the associated computational burden. In addition, in our setting, the interaction terms would involve continuous variables. Interaction between two continuous variables are often difficult to interpret in a biologically meaningful way, which further limits their practical utility in this context. We also clarify in the Discussion that extending the framework to accommodate non-linear or interaction effects in a computationally feasible manner remains an important topic for future work.

Specifically, we added the following sentence to the Discussion:
“Future work may consider extensions incorporating non-linear or interaction effects. Although feasible in principle, such extensions would substantially increase computational burden in high-dimensional settings, and interactions between continuous variables may be difficult to interpret biologically, potentially limiting their practical utility.”

Furthermore, regarding the “proportional effect assumption,” we would like to clarify that since our method is based on an accelerated failure time (AFT) model, the proportional effect assumption (which is usually for Cox model) is not required for our method.

2. Simulated data design is overly idealized and inconsistent with the characteristics of real-world data: In the simulation experiments, exposure variables (X) were set to follow a multivariate normal distribution with no correlations, and the association patterns of mediator variables (M) (e.g., 40% of M associated with 10% of X) were artificially predefined. This differs significantly from the characteristics of real multi-omics data (e.g., high co-expression among genes, heterogeneous associations between proteins and genes, and batch effects). Such an idealized design may overestimate SMAHP’s performance in real-world data. For example, if strong collinearity exists among exposure variables in real data, the variable screening performance of MCP-penalized regression may decline, but this scenario was not simulated. It is recommended to supplement simulation settings that better mimic real multi-omics data (e.g., introducing variable correlations, batch effects, and outliers) and re-validate SMAHP’s performance to ensure the method’s reliability in complex data scenarios.

**Authors’ response**: We sincerely thank the reviewer’s comment and the thoughtful suggestions regarding the realism of the simulation design. We have now conducted sensitivity analyses that incorporate correlated gene and protein structures, please see our responses to the first reviewer’s comments 1 and 2 for more details about correlated genes and proteins.

We also appreciate the reviewer’s suggestion to supplement simulation settings with outliers, which are now included in additional sensitivity analyses (provided as in S4 Table). Regarding batch effects, while they are an important consideration in practical multi-omics studies, modeling batch-specific technical variation requires assumptions and preprocessing strategies that are highly data- and platform-dependent. As our primary objective is to evaluate the statistical properties of the proposed mediation framework under controlled data-generating mechanisms, explicitly modeling batch effects is beyond the scope of the current study.

3. Real-world data validation lacks depth and breadth: The current application is limited to single-cohort CPTAC head and neck squamous cell carcinoma (HNSCC) data (final sample size: 102 patients, after excluding 7 patients with missing overall survival (OS) data). External validation using multi-center, multi-disease data is lacking, making it difficult to demonstrate SMAHP’s generalizability. For example, the finding that "LCE3E mediates the effect of HMGB1P23 on OS" was not validated in other cancer types (e.g., lung cancer, breast cancer) or independent HNSCC cohorts, leaving open the possibility that this pathway is a cohort-specific occasional result. Furthermore, the application study did not extend the analysis to meet clinical practice needs—for instance, it did not explore the association between the mediation pathway and treatment response (e.g., chemotherapy sensitivity), nor did it validate the model’s performance in real-world data with small sample sizes or high censoring rates. It is recommended to supplement external validation with multi-cohort data (e.g., TCGA data, independent clinical study data), conduct analyses on multi-omics data from different disease types, and deepen result interpretation by integrating clinical indicators to enhance the method’s practical application value.

**Authors’ response**: We thank the reviewer for the thoughtful comment regarding the depth and breadth of real-world data analysis. We acknowledge that the manuscript does not aim to provide external validation in the sense of replicating identical mediation findings across independent cohorts or disease types which will be important future research projects. However, we were able to validate the result of the real study application by using a logistic error term (instead of a normal error term) in the AFT model, which also shows the robustness of our approach.

We also conducted additional exploratory analyses in other disease settings, including TCGA pancreatic cancer data and CPTAC pancreatic cancer data. In these analyses, we identified one significant gene-protein-survival pathway in the CPTAC pancreatic cancer data (ELAPOR1 gene-PLOD1 protein on survival; p-value = 0.0001) but did not identify any significant gene-protein-survival pathway in the TCGA pancreatic cancer data. These additional analyses were intended to assess the general applicability of the proposed method to other real-world datasets rather than to draw definitive biological conclusions. The limited findings are likely attributable to the relatively small sample sizes of these datasets (97 and 113 samples for CPTAC and TCGA pancreatic cancer data, respectively), and larger cohorts may be required to identify additional pathways.

4. Robustness of causal inference assumptions is not fully demonstrated: The core assumption of causal mediation analysis—"no unmeasured confounding" (i.e., no omitted confounders affecting the associations between exposure and outcome, mediator and outcome, or exposure and mediator)—was not validated. The authors did not discuss potential unmeasured confounders in the CPTAC data (e.g., patient smoking history, alcohol consumption, comorbidities), nor did they perform sensitivity analyses (e.g., E-value calculation, simulating the effects of unmeasured confounders) to assess the impact of these factors on mediation effect estimates. Additionally, the study did not consider "interactions between mediators". In real biological systems, proteins often exhibit regulatory relationships, but SMAHP did not include such interaction terms, potentially missing key causal pathways. It is recommended to supplement sensitivity analyses to validate the rationality of the "no unmeasured confounding" assumption, or extend the analysis by incorporating mediator interaction terms into the model to improve the rigor of causal inference.

**Authors’ response**: Thank you for the comment. We agree that sensitivity analyses is an important way to assess the impact of “unmeasured confounding” in general. Regarding the sensitivity analysis using E-values^1^, we attempted to compute E-values using the *EValue* R package based on effect estimates from the final outcome model. Although the package documentation indicates that the “evalue()” function can be applied to log-linear models, it still requires specifying an effect measure (e.g., OLS-based measures). We therefore tried to evaluate the feasibility of using OLS-based effect measures; however, we found this to be inappropriate for the mediator-outcome assessment in our setting, as the outcome is modeled using an accelerated failure time framework with censoring. Since OLS-based effect measures do not properly account for censoring, E-value analyses were not included. Regarding simulating the effects of unmeasured confounders, that is a very important aspect of sensitivity analyses^2^ which could be a future project focusing on sensitivity analyses rather than focusing on modeling.

As stated in the response to the above comment 3, we were able to validate the result of the real study application by using a logistic error term (instead of a normal error term) in the AFT model, which also shows the robustness of our approach.

Please also see the response to the above comment 1 regarding the robustness of our approach under some other assumptions as well as incorporating interaction terms.

References:

[1] Smith LH, VanderWeele TJ. Mediational E-values: Approximate Sensitivity Analysis for Unmeasured Mediator-Outcome Confounding. Epidemiology. 2019 Nov;30(6):835-837.

[2] VanderWeele TJ. Mediation Analysis: A Practitioner's Guide. Annu Rev Public Health. 2016;37:17-32.

Minor Comments
1. It is recommended to supplement the simulation experiments with an analysis of "the impact of variable dimensionality (p/k) on computational time" to provide readers with references for computational cost when selecting the method.

**Authors’ response**: In main tables (Tables 1-3), we report the average computational time under varying parameter configurations, including different levels of variable dimensionality (p and k), sample size (n), and censoring rates. In addition, the Simulation Results section specifies that all simulation experiments were conducted using 10 CPU cores and 8 GB of RAM per node, providing details on the computational resources required to run the proposed method.

2. Figure 1 (causal pathway diagram) could be supplemented with 95% confidence intervals to intuitively illustrate the uncertainty of effect estimates.

**Authors’ response**: Thank you for your thoughtful suggestion. We have updated Figure 1 with 95% confidence intervals for each effect estimates.

3. The Discussion section should explicitly propose optimization directions for SMAHP in "scenarios with explosive growth in multi-omics data volume" (e.g., integration of parallel computing, dimensionality reduction algorithms) to provide insights for future research.

**Authors’ response**: We thank the reviewer for this helpful suggestion. In response, we have revised the Discussion to more clearly outline potential directions for improving the computational performance of SMAHP in settings with rapidly increasing multi-omics data dimensionality. In particular, the revised Discussion highlights computational strategies that may facilitate the application of SMAHP to increasingly high-dimensional multi-omics data. Below is what we added in the end of Discussion.

“From a computational perspective, further improvements may also be achieved by leveraging parallel computing strategies and incorporating dimensionality reduction or feature screening steps such as marginal screening to reduce computational complexity when analyzing increasingly high-dimensional multi-omics data.”

**Reviewer #3**

**Reviewer #3:** The authors propose the SMAHP framework, which is a novel designed for high-dimensional causal mediation analysis integrating genomic, proteomic, and survival data. This addresses an important gap in existing methods which focus on single exposures, limited applicability to complex multi-omics data where multiple genes may work through multiple proteins to affect survival. The proposed three-step approach combining penalization, sure independence screening, and hypothesis testing is computationally feasible and demonstrates good performance in simulations under the conditions tested. The approach is technically sound, and the paper is generally well-written. However, the current manuscript would be strengthened by addressing several issues:

**Authors’ response**: Thank you for the appraisal.

1. The authors indicate that the exposure variables (genes) are not correlated. By simulating independent genes, the authors test their method under a best-case scenario that likely overestimates performance. When genes are correlated, penalization methods like MCP would have to choose among correlated signals. This could lead to unstable variable selection and difficulty determining which gene is the true mediator versus simply correlated with it. Have the authors examined correlations among the top 100 genes selected from CPTAC data? If HMGB1P23 is highly correlated with other top-ranked genes, how confident can we be in the specificity of this finding?

**Authors’ response**: We appreciate the reviewer’s comment. In the CPTAC study, we calculated pairwise correlations between HMGB1P23 and other genes among the top 100 genes selected after the screening step. The third quartile and maximum of the magnitudes of observed correlations were 0.284 and 0.487 respectively, which do not indicate strong collinearity among the selected genes. In addition, we conducted sensitivity analyses incorporating correlated gene and protein structures which showed consistent performance. Please see the responses to the first reviewer’s comments 1 and 2 for more details.

2. The authors specifically select 100 genes and 200 proteins from pre-screening for SMAHP. Is the selection based on computational constraints or other considerations? Given that the starting dataset contains 60,669 genes and 9,469 proteins, reducing to 100 genes and 200 proteins seems to be a stringent threshold and could lose a true signal ranked just outside these cutoffs. Sensitivity analyses by repeating the analyses with alternative cutoffs could be helpful.

**Authors’ response**: We thank the reviewer for this thoughtful comment. The choice of retaining 100 genes and 200 proteins after pre-screening was guided primarily by the sample size of the CPTAC-HNSCC dataset (n = 102) and the need to balance model complexity with statistical power. Including a substantially larger number of features in a high-dimensional mediation framework can lead to unstable estimation and reduced power. We acknowledge that alternative pre-screening cutoffs may retain additional signals. However, in practice, substantially increasing the numbers of genes and proteins retained at the screening stage led to numerical instability and convergence failures in the SMAHP fitting procedure due to the extreme dimensionality relative to the sample size. As a result, we did not pursue formal sensitivity analyses with larger cutoffs, and instead focused on thresholds (100 genes and 200 proteins) that allow stable model estimation in this setting.

3. Based on my understanding, the method tests each protein mediator independently. However, proteins could have multiple weak sequential effects that combine to create a strong overall pathway. A gene can influence one protein and subsequently activates another protein, then affects survival. SMAHP tests each link separately and may find each individual effect too weak to reach significance even though the complete cascade has a substantial cumulative effect on survival outcome. While this limitation is shared by some other high-dimensional methods, the authors could further clarify this as a current limitation.

**Authors’ response**: Thanks for the insights. We have added the acknowledgement of that limitation in the discussion section as follows:

“A typical limitation of high dimensional mediation modeling methods is that it cannot disentangle the potential sequential causal mediation effect through multiple mediators due to the complexity induced by the high dimensionality of mediators. As with many existing methods, our proposed method does not test any sequential causal mediation effects or the cumulative causal mediation effect of multiple mediators.”

4. The authors mentioned that the proposed method identified a gene-protein pair associated with survival, specifically HMGB1P23 and LCE3E. To further elucidate the biological relevance of the identified HMGB1P23–LCE3E–survival pathway, the author could conduct additional analyses? For example, examining the correlation between HMGB1P23 gene expression and LCE3E protein abundance? Stratified survival analyses across joint expression strata of HMGB1P23 and LCE3E might also help to understand whether the mediation pattern translates into distinct survival outcomes. It would also be helpful to evaluate the robustness of the findings with other tests, which would strengthen confidence in the observed HMGB1P23–LCE3E mediation effect.

**Authors’ response**: Thank you for the comment. To further explore the relationship between the identified gene-protein pair, we examined the correlation between HMGB1P23 gene expression and LCE3E protein abundance. The correlation coefficient was 0.488 (p $=1.98\times{10}^{-7}$), indicating a strong positive association.

We also conducted survival analyses using log-rank tests. Specifically, we categorized HMGB1P23 gene expression and LCE3E protein abundance into quintiles (five groups) and evaluated their marginal associations with survival using a multi-group log-rank test. The log-rank test yielded p=0.001 for HMGB1P23 gene expression and p=0.03 for LCE3E protein abundance.
