# Supplementary material for: A multi-omics framework for survival mediation analysis of high-dimensional proteogenomic data
Source: PLoS Comput Biol. 2026 Apr 27;22(4):e1014217. doi: 10.1371/journal.pcbi.1014217 (PMC13138757; doi:10.1371/journal.pcbi.1014217)
Supplement: Attachment — Submitted filename: Point_to_Point_Response.docx [file pcbi.1014217.s012.docx]

**Dear Editor:**

Thank you for your thoughtful suggestion in the decision letter to include a Data and Code Availability section in the main body of the manuscript (Page 16; Lines 477–482). We have revised the manuscript accordingly and added the section as below. There is also a sentence about the R package availability in Abstract:

“**Data and Code Availability**

The pre-processed RNA-Seq, proteomics, and clinical data (metadata) from the National Cancer Institute-initiated CPTAC are available in the Proteomic Data Commons (https://pdc.cancer.gov/pdc/cptac-pancancer). The SMAHP (all upper cases) R package is freely available in the Comprehensive R Archive Network (CRAN) repository (https://cran.r-project.org/web/packages/SMAHP/index.html).”

We removed “Please reach out to the corresponding author (Seungjun Ahn, seungjun.ahn@mountsinai.org) if you have any further inquiries about the R package” since the journal technical check informed not to include the point of contact in the section. In addition, we tested PLOS’s NAAST tool and updated all figures to TIF format, which have been uploaded with this resubmission.

Thank you again for your consideration.
